# A high-throughput microfluidic approach for 1000-fold leukocyte reduction of platelet-rich plasma

**DOI:** 10.1038/srep35943

**Published:** 2016-10-24

**Authors:** Hui Xia, Briony C. Strachan, Sean C. Gifford, Sergey S. Shevkoplyas

**Affiliations:** 1 Department of Biomedical Engineering, University of Houston, Houston, TX 77204, USA

## Abstract

Leukocyte reduction of donated blood products substantially reduces the risk of a number of transfusion-related complications. Current ‘leukoreduction’ filters operate by trapping leukocytes within specialized filtration material, while allowing desired blood components to pass through. However, the continuous release of inflammatory cytokines from the retained leukocytes, as well as the potential for platelet activation and clogging, are significant drawbacks of conventional ‘dead end’ filtration. To address these limitations, here we demonstrate our newly-developed ‘controlled incremental filtration’ (CIF) approach to perform high-throughput microfluidic removal of leukocytes from platelet-rich plasma (PRP) in a continuous flow regime. Leukocytes are separated from platelets within the PRP by progressively syphoning clarified PRP away from the concentrated leukocyte flowstream. Filtrate PRP collected from an optimally-designed CIF device typically showed a ~1000-fold (i.e. 99.9%) reduction in leukocyte concentration, while recovering >80% of the original platelets, at volumetric throughputs of ~1 mL/min. These results suggest that the CIF approach will enable users in many fields to now apply the advantages of microfluidic devices to particle separation, even for applications requiring macroscale flowrates.

A microliter of whole blood (WB) contains approximately 5000 to 10000 leukocytes – in order to fight infection and regulate the body’s immune response. For patients receiving transfusions of donated blood products, however, leukocytes are an undesirable contaminant. There is ample evidence suggesting that allogeneic leukocytes contribute to a number of transfusion-related complications, including febrile non-haemolytic transfusion reactions, transmission of cytomegalovirus and variant Creutzfeldt-Jakob disease, post-transfusion alloimmunization, and transfusion-related acute lung injury^1^,^2^. Clinical studies have shown that ‘leukoreduction’ (LR) of donated blood components reduces these risks significantly^3^,^4^,^5^,^6^, and therefore universal leukoreduction of all blood products intended for transfusion is now mandated in most developed countries^2^,^7^.

While the technical definition of leukoreduction varies slightly among different areas of the world, in general, the concentration of leukocytes must be less than just 10/μL for the blood product to be marketed as ‘leukoreduced’ – an approximate 1000-fold reduction in leukocyte concentration from the original level in the blood of the donor^8^. Currently, leukoreduction for WB-derived components is performed using LR filters that physically retain leukocytes (either due to electrostatic attraction to the fibres of the filter, or by being mechanically trapped within the tortuous pores of the filtration material), while allowing the desired blood product(s) to pass through^9^,^10^,^11^. Driven by gravity, or a spring-loaded plasma expressor, this method typically will process a 200–250 mL unit of platelet rich plasma (PRP) in about 10 min, while removing more than 99.9% of the leukocytes and recovering 80–90% of the platelets originally contained in the unit^8^.

Conventional LR filters, even when operating properly, expose the blood product filtrate to trapped leukocytes (which are typically activated by the interaction with the filtration material) for the duration of the leukoreduction procedure. Further, the utility of LR filters can be limited by their tendency to progressively clog with the leukocytes retained during filtration^12^,^13^. Consequently their use has been associated with the release from leukocytes of cytokines and microparticles (which can trigger a harmful inflammatory response in the transfusion recipient)^14^,^15^,^16^, as well as increased platelet activation (which can lower platelet post-transfusion efficacy and viability)^16^,^17^.

A microfluidics-based approach could potentially circumvent these fundamental limitations of conventional LR filters by enabling continuous separation of leukocytes from a stream of WB or PRP, at the micro-scale^18^,^19^. Removal of leukocytes without physical trapping would prevent the release of potentially harmful cytokines into the blood product, and maintain the efficiency of leukoreduction over time. One possible approach to achieving leukoreduction of PRP using microfluidics is to exploit the substantial difference in size between leukocytes (7–15 μm) and platelets (1.5–4.0 μm). Numerous microfluidic techniques for size-based particle separation have been developed, including deterministic lateral displacement (DLD)^20^,^21^,^22^,^23^, cross flow filtration^24^,^25^,^26^,^27^,^28^, biomimetic separation^29^,^30^, inertial flow^31^,^32^, and pinched flow fractionation^33^. These techniques typically require complex fabrication processes and/or small device feature sizes, and cannot reliably separate blood cells with sufficient purity and throughput to enable practical leukoreduction of WB or diluted WB. (The removal of leukocytes from PRP, however, is a relatively less complex task, and therefore some of these technologies may show improved performance when processing this simpler type of input sample).

Our group has recently developed a novel microfluidic approach for size-based particle/cell separation, referred to as ‘controlled incremental filtration’ (CIF). Unlike other microfluidic separation techniques which utilize simple size-exclusion (i.e. with exceedingly small ‘sieves’ or ‘filtration pores’), the CIF approach allows for the separation of particles that are substantially smaller than the minimum feature size of the device. With a relatively large minimum feature size (i.e. ~20 μm), the CIF approach enables fabrication of deeper devices (i.e., ≥140 μm, when using standard soft lithography) than many of the aforementioned microfluidic methods. Larger device dimensions enable a higher volumetric flowrate (or throughput) at a given driving pressure, and reduce the probability of device clogging, while generally imposing less shear stress on the cells being processed at a given flowrate. Here, we describe the development and validation of a CIF-based microdevice capable of high-throughput leukoreduction of platelet rich plasma, which overcomes the long-standing limitations of conventional filters and existing microfluidic methods alike.

## Results

### Design of next-generation controlled incremental filtration (CIF) devices

**Figure 1:**
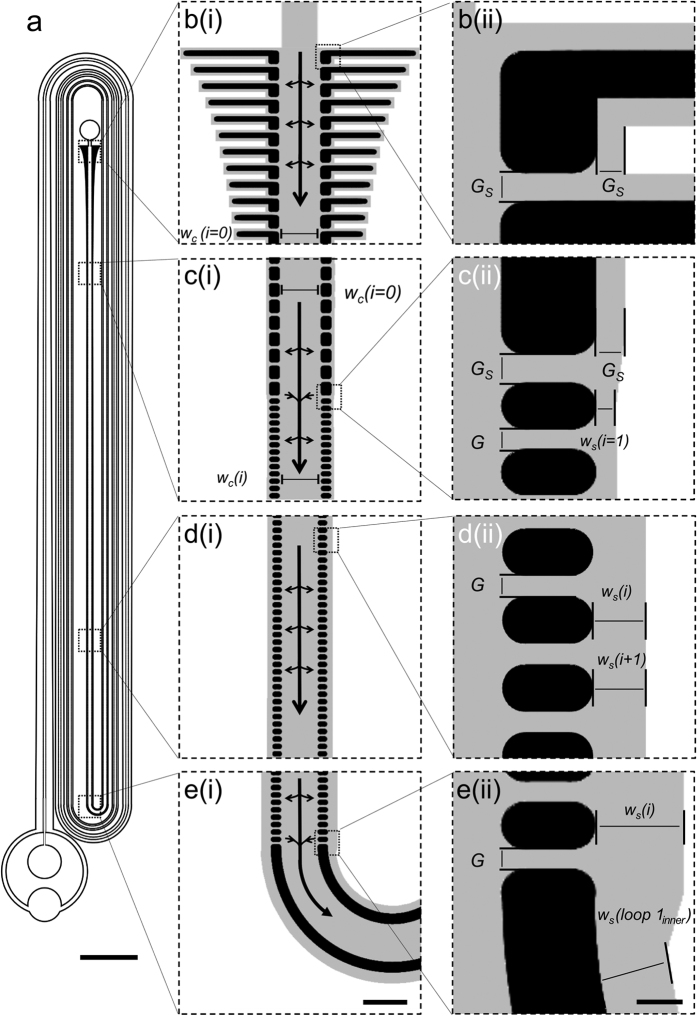
Design of a reduced shear (RS) CIF device (c.d. = 7 μm). (**a**) Overall layout of the device. (**b**) Device inlet: (i) the initial width of the centre channel is *w*_*c*_(*i* = *0*) = 300 μm; the channel is flanked by a series of serpentine side segments; (ii) the width of the side segments, *G*_*S*_ = 22.8 μm, is set slightly larger than the width of the subsequent inter-post gaps, G = 16 μm. (**c**) Side channel architecture transition: (i) progressively-shortening serpentine segments ultimately become rectilinear side channels, after which the width of the centre channel *w*_*c*_(*i*), initially constant, progressively narrows; (ii) the width of the side channels at the first set of pill-shaped posts, *w*_*s*_(*i* = *1*), is set to equal the desired width of the inter-post gap, i.e. the smallest feature of the device. (**d**) Central and side channel progression: (i) the width of the central channel, *w*_*c*_*(i)* gradually decreases until it reaches 150 μm, and remains constant thereafter; (ii) the width of the side channels continues to gradually increase, *w*_*s*_(*i* + *1*) > *w*_*s*_(*i*), throughout the remainder of the device. (**e**) Loop transition: (i) the first leg of device transitions into the first loop; (ii) to maintain appropriate fluidic streamlines, the width of the inner side channel decreases from *w*_*s*_(*i*) = 69.5 μm to *w*_*s*_(*loop 1*_*inner*_) = 57.6 μm; correspondingly, the width of the outer side channel increases to *w*_*s*_(*loop 1*_*outer*_) = 77.4 μm (not shown). Arrows indicate direction of fluid flow. Scale bars: (**a)**, 5 mm; (**b–e)(**i), 250 μm; (**b–e)**(ii), 50 μm.

The mathematical framework of the CIF approach has been previously described in detail. This approach enables one to quickly design microfluidic devices that can selectively concentrate particles above a certain size (or ‘critical diameter’, c.d.) to a high degree (e.g. 10-fold and higher), without relying on the ultrafine microfabrication methods that inherently limit the manufacturability and volumetric throughput of other microfluidic strategies. The CIF approach uses a recursive calculation to generate the pattern of a ‘co-flow’ device in which a centre channel retains particles that are larger than the desired c.d., and two adjacent ‘side channels’ which progressively increase in width along the length of the device. The rate of this increase determines the fraction of fluid flow, *f*_*gap*_, that is syphoned away from the centre channel at each ‘gap’ in the two sets of ‘posts’ that separate the centre and side channels. The larger the value of *f*_*gap*_, the larger the c.d. of a CIF-based device. Once the desired value of *f*_*gap*_ (and dimensions of the centre channel, posts, and gaps) are selected, a CIF-based device can be rapidly patterned numerically, and structured into a paperclip-like format (Fig. 1a) to achieve the preferred amount of particle concentration/filtration in a compact footprint. In this study we refined the CIF approach to further increase the minimum feature size, ensure lossless fluidic transitions within the paperclip-like format, and minimize shear experienced by particles in the device.

#### Modification of the initial section of side channels

The original CIF publication described the initial width of a device’s side channels to be a small, finite value, which would in practice be limited by the minimum achievable feature size of the fabrication method. We eliminated this limitation by replacing the narrow initial section of the side channels with a series of much wider serpentine segments of equivalent fluidic resistance, flanking the centre channel on both sides (Fig. 1b(i)). The width of the serpentine segments remains constant and their length progressively decreases along the length of the device, reducing their effective fluidic resistance in the same manner that a progressive increase in side channel width, *w*_*s*_(*i*), does, in order to draw incrementally more fluid out of the centre channel of the device. That is, the designated value of *f*_*gap*_ determines how quickly their length decreases, using the same mathematical framework that governs the subsequent increase in *w*_*s*_(*i*).

The width of the serpentine segments, *G*_*S*_, is selected to be slightly larger than the gap size, *G* (Fig. 1c(ii)). By choosing this value appropriately, one can ensure that there is minimal fluidic mixing when these segments then transition to the progressively-widening side channels, following the initial section of a CIF device (Fig. 1c(i,ii)). In this study, the gap size of the device, *G*, was 16 μm, and the width of the serpentine segments, *G*_*S*_, was chosen to be 22.8 μm. These design choices cause the fluid in the side channels to be pulled into the centre channel slightly at the transition (Fig. 1c(i,ii)), and thus ensure that cells of interest were not lost to the side channels. Following the transition area (Fig. 1d(i,ii)), *w*_*s*_(*i*) grows continuously until the desired final side channel width is reached, and this endpoint is determined by the degree of particle concentration (or, equivalently, the amount of filtration) preferred for a given application. The minimum feature size for a CIF device designed according to these principles is now equivalent to its gap size, *G,* which is defined by the requirements of the application and other design criteria, rather than what is achievable using the available fabrication method.

#### Ensuring lossless fluidic transitions within the paperclip-like format of a CIF device

The simple, recursive nature of the CIF approach enables patterning devices of any total length that is needed to produce the degree of particle enrichment/separation demanded by a particular application. In practice, however, one must partition a lengthy CIF device into ‘legs’ in order to fit its overall footprint onto a standard substrate mould, e.g. a 3′′ or 4′′ silicon wafer. At the end of each leg of a device, a set of semi-circular channels serves to wrap its centre and side channels back 180° (Fig. 1e(i,ii)), ultimately resulting in the paperclip-like format of our complete devices (Fig. 1a). These ‘loop’ channels are designed similarly to the aforementioned transition area between the initial serpentine segments and the subsequent progressively-widening section of the side channels (Fig. 1c(i,ii)), in that the widths of the side channel loops are lowered slightly from their mathematically-predicted values to ensure that the particles of interest are retained in the centre channel as fluid flows from leg to leg of the device. In practice, one can simply subtract 1–5 μm from the predicted side channel widths (of the loops themselves, as well as the downstream side channels of the next device leg), to effect a slight ‘pulling in’ of fluid into the centre channel. The ideal amount of the adjustment will depend upon the width and length of the channels at a given loop, as well as the user’s preference for how aggressively to pull fluid back into the centre channel weighted against the associated loss in overall efficiency of the device.

#### Reduction of shear within CIF devices

The final refinement of the CIF approach we introduced in this study was made in order to reduce the maximum amount of shear stress experienced by particles within the device. In the original CIF approach (OR-CIF), the width of the centre channel, *w*_*c*_(*i*), would remain constant while the side channels progressively widened, resulting in a higher level of shear experienced near the inlet of the device. In the next-generation reduced-shear CIF approach (RS-CIF), we begin with a wider centre channel, which progressively narrows (as the side channels widen) along the length of the device. The degree of centre channel narrowing relative to the degree of side channel widening at each filtration gap, *i*, in an RS-CIF device is determined numerically (using formulas for rectangular channels based on Yang *et al.*) in order to maintain a relatively constant amount of (maximum) shear experienced along each leg of the device, while still satisfying the recursive framework constraints of OR-CIF^34^.

**Figure 2:**
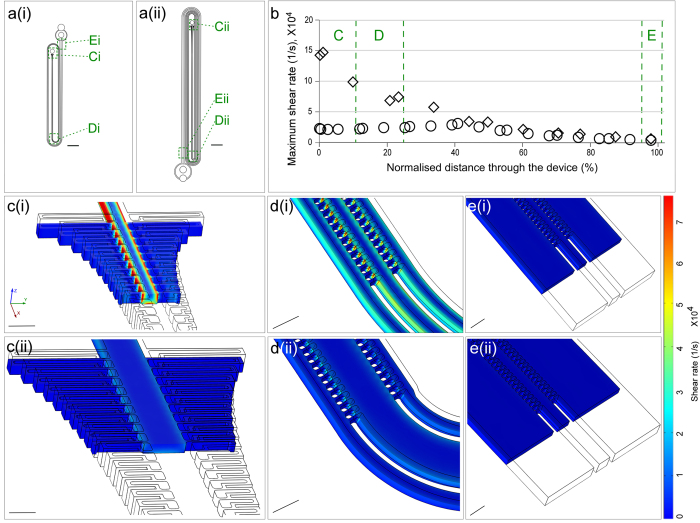
Estimated shear rates within original (OR) and reduced shear (RS) CIF devices (c.d. = 7 μm), under a driving pressure of 25 PSI. (**a**) Schematics of the (i) OR-7 and (ii) RS-7 devices. (**b**) Maximum shear in a given device [OR-7, diamonds; RS-7 circles] segment typically occurs on the post surface that faces the centre channel. (**c–e**) Results of simulations in COMSOL Multiphysics illustrating the shear rate in the two devices at their inlet area, first loop, and channel outlets, respectively. Each image shows colour-scaled surface shear values of the cross-sectioned device segments at their centre plane and below (i.e. z ≤ 74.5 μm), with a wireframe rendering of the balance of the design. Scale Bars: (**a)**, 5 mm; (**c–e**), 200 μm.

As illustrated in Fig. 2, the OR- and RS-CIF device designs with an approximately-equivalent c.d. of ~7 μm (“OR-7”, Fig. 2a(i), and “RS-7”, Fig. 2a(ii)) were modelled using three-dimensional computational fluid dynamics (CFD) software to compare differences in the shear rates experienced by particles that travel through these devices. The CFD simulations showed that the maximum shear rate near the entrance of the RS-7 device (~2.2 × 10^4^/s at 25 PSI) was significantly lower than that of the OR-7 (~14 × 10^4^/s), due to the difference in initial centre channel widths (Fig. 2b, also compare Fig. 2c(i) and Fig. 2c(ii). The shear rate in the first half of the RS-7 device remained approximately constant before falling once the minimum centre channel width was reached (Fig. 2b), as after this point the centre channel flow velocity begins to slow. There was a slight increase in shear within the centre channel immediately downstream of each loop transition (Fig. 2b), due to the deliberate step-down in side channel width of those areas (described above).

### Performance of the CIF-based devices for leukoreduction of PRP

**Figure 3:**
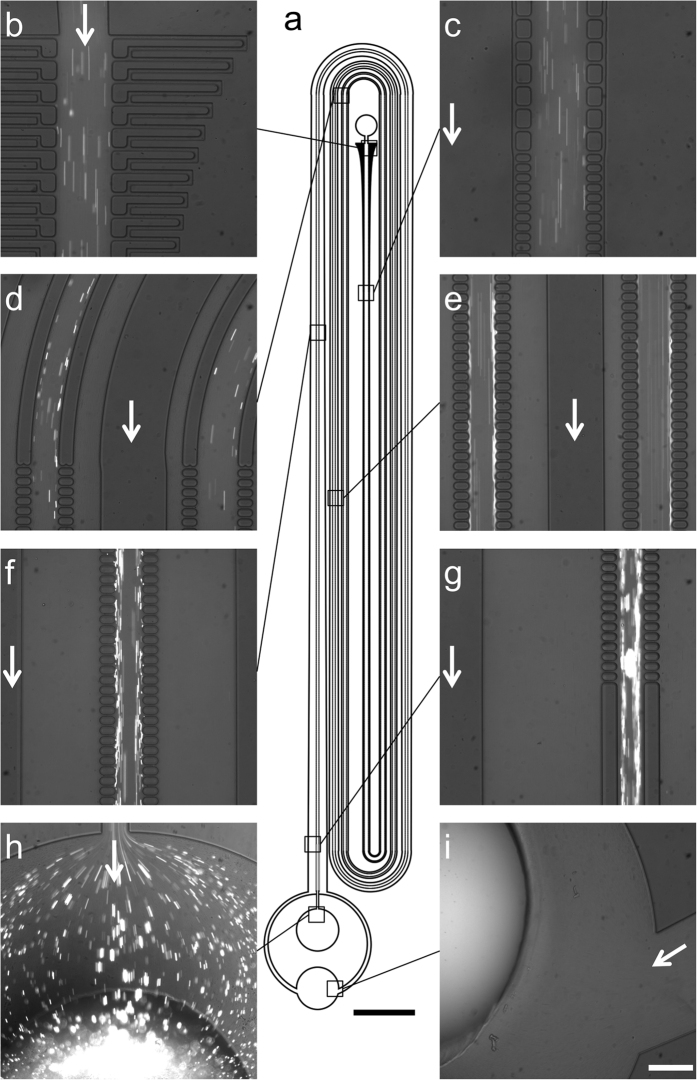
Images of an RS-CIF device performing leukocyte reduction of PRP. (**a**) Schematic illustration of the RS-7 device. (**b-i**) Fluorescent images of DNA-stained leukocytes within a PRP sample as they are progressively concentrated in the centre channel, while flowing through the device: (**b**) device inlet; (**c**) side channel architecture transition; (**d**) end of the second (right) and fourth (left) loop of the device; (**e**) the third (right) and fifth (left) legs of the device; (**f**) the seventh leg of the device; (**g**) device outlet; (**h**) central channel (retentate) collection port for highly-concentrated leukocytes; **(i)** side channel (filtrate) collection port for leukocyte reduced PRP. Filtrate-to-retentate volumetric flow ratio is approximately 15:1. Scale Bars: (**a**), 5 mm; (**b–i)**, 250 μm.

Figure 3, Supplementary Video 1, and Supplementary Figure 1 illustrate operation of CIF devices performing leukoreduction of PRP (although all of these images were acquired while using a driving pressure lower than in typical experiments, to enable clear visualization of fluorescently-labelled leukocytes). Concentration of leukocytes in the centre channel of the device is visually apparent (compare Fig. 3b,g), with almost no leukocytes escaping to the side channels (compare Fig. 3h,i) as ~93.8% (corresponding to a concentration factor of ~15×) of the total volume of PRP originally input into the device is filtered from the centre to the side channels.

**Figure 4:**
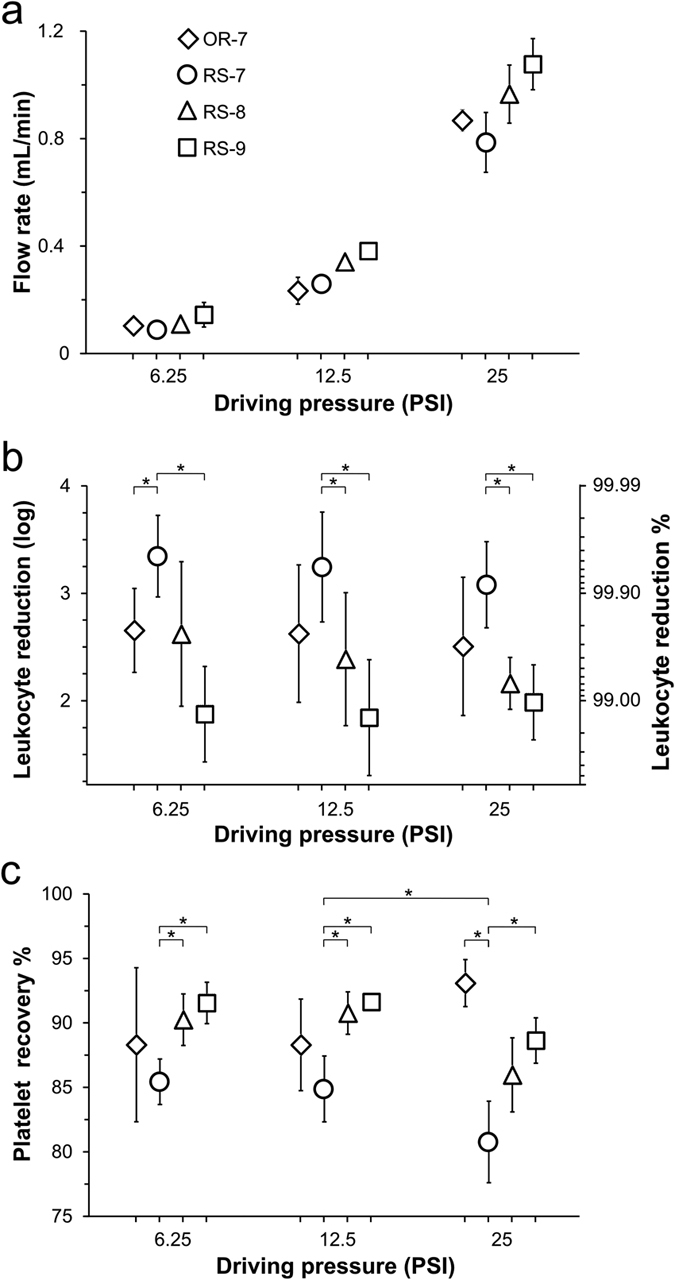
Performance of OR-7, and RS-7/8/9 CIF devices at driving pressures of 6.25, 12.5 and 25 PSI. (**a**) Volumetric throughput. (**b**) Leukocyte reduction (in log depletion and percent depletion) of collected filtrate relative to input PRP sample. (**c**) Platelet recovery in filtrate. All values shown as mean ± s.d. (n = 5). Asterisks represent a significant difference (p < 0.01) between different devices at a given driving pressure or a given device at different driving pressures.

We tested both the OR-7 and RS-7 devices with freshly-donated PRP to determine the extent of leukocyte reduction, platelet recovery, and platelet activation associated with each design. We also tested two additional RS-CIF devices with nominal c.d. of 8 μm (“RS-8”, *w*_*c*_(*ref*) = 100 μm and *f*_*gap*_(*ref*) = 7.2 × 10^−4^) and 9 μm (“RS-9”, *w*_*c*_(*ref*) = 100 μm and *f*_*gap*_(*ref*) = 8.6 × 10^−4^) to study the effect of the value of c.d. on these performance parameters. Figure 4 summarizes the results of these studies.

The relationship between the driving pressure and flowrate for all devices was not linear, indicating the presence of pressure-induced deformations of the device architecture (Fig. 4a). The OR-7 device was able to produce, on average, a 2.7-log reduction of leukocyte concentration at 6.25 PSI, and the device performance declined slightly with increasing pressure down to 2.5-log leukoreduction at 25 PSI. The performance of the RS-7 device also declined slightly with increasing pressure, but was consistently better as the device was able to accomplish a greater than 3-log (i.e. >99.9%) reduction of leukocyte concentration in the PRP for all pressures studied (Fig. 4b). This improvement was likely due to a combination of the larger centre channel of the RS-7 device, and the aforementioned refinements of the modelling framework used to generate its microchannel architecture, which minimized the effect of PDMS deformation at higher pressures (Supplementary Videos 2 and 3). As expected, the RS-8 and RS-9 devices showed progressively lower efficacy of leukocyte removal than either OR-7 or RS-7, as increasing the c.d. of the device allows more leukocytes to escape from the centre to the side channels.

All three RS devices showed a decrease in platelet recovery with increasing driving pressure: from 85.4% to 80.8% for RS-7, from 90.2% to 86.0% for RS-8, and from 91.5% to 88.6% for RS-9 as the driving pressure increased from 6.25 PSI to 25 PSI (Fig. 4c). In contrast, the platelet recovery for the OR-7 device showed an opposite trend (increasing from 88.3% to 93.1%), which suggests that the narrower geometry of the OR-7 device was likely more prone to pressure-induced deformation (causing an increase in its effective value of *f*_*gap*_) than were the wider next-generation RS devices.

**Figure 5:**
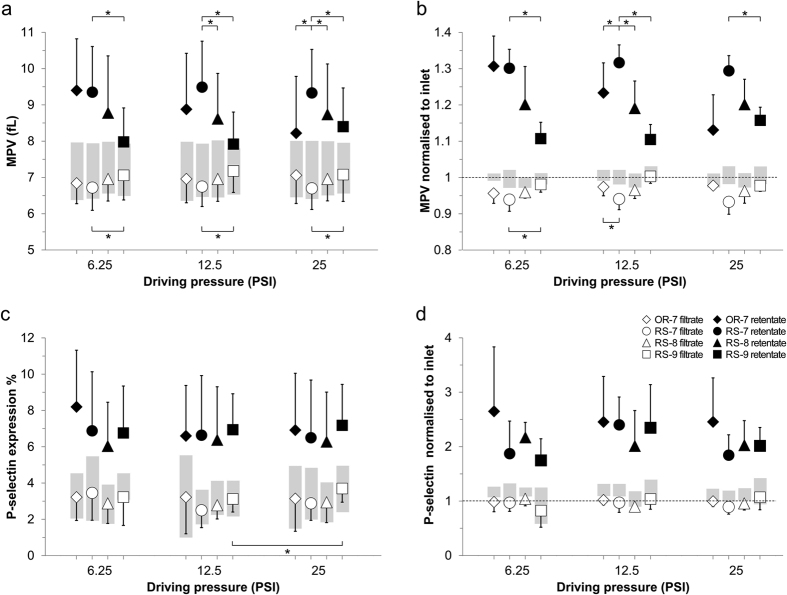
Platelet metrics for leukocyte reduced PRP (filtrate; open symbols) and leukocyte concentrated PRP (retentate; filled symbols) output from CIF devices. Plots of: (**a**) absolute MPV values, (**b**) MPV normalized to the corresponding inlet sample, (**c**) platelet P-selectin expression, and (**d**) normalized P-selectin expression; shown as mean ± s.d. (n = 5) for the four CIF devices under different driving pressures. Outlet sample data for the OR-7, RS-7, RS-8, and RS-9 devices are represented by diamonds, circles, triangles, and squares, respectively. Retentate measurements in all plots are significantly higher than corresponding filtrate data (p < 0.05). Grey boxes represent mean ± s.d. of: (**a**,**c**) the inlet sample measurements, or (**b**,**d**) the cumulative retentate and filtrate values, weighted by their respective platelet recovery. Dotted lines (**b**,**d**) represent the normalized inlet values, by definition equal to unity. Average filtrate results show slightly lower MPV (in all 9 data points; B) and P-selectin expression (in 6 of 9; (**d**)) than corresponding inlet values of the three devices found best suited to perform leukocyte reduction (i.e. OR-7, RS-7, and RS-8). Cumulative weighted MPV data show no significant difference with inlet MPV, while cumulative weighted P-selectin data are on average ~10% higher than the corresponding inlet values; indicating a small degree of additional platelet activation in the retentate channel but no increased platelet aggregation in either channel. Asterisks represent significant difference (p < 0.01) in paired data between the devices or driving pressures indicated.

Figure 5 shows the effects of passing through a CIF device on the mean platelet volume (MPV) and platelet activation (quantified via P-selectin expression) for platelets recovered from the centre (retentate) and side (filtrate) outlets of the devices. The MPV values for platelets from the retentate were lower (and from the filtrate – higher) for devices with larger c.d., because larger values of c.d. allowed larger platelets, and existing platelet aggregates, to be pulled into the side channels (Fig. 5a). There was little change in MPV values for the RS-7 and RS-8 devices with increasing driving pressure (Fig. 5a). The MPV of platelets in the retentate for the OR-7 device decreased with increasing driving pressure (Fig. 5a), mirroring the device’s increase in platelet recovery (Fig. 4c). The retentate MPV of the RS-9 device increased slightly for the highest driving pressure (Fig. 5a), which agrees well with more leukocytes being retained in the centre channel of the RS-9 device at higher driving pressures (Fig. 4b). It is important to note that MPV may not be a reliable measure of a device’s particle separation performance, as platelets can quickly aggregate, particularly when exposed to the high shear rates common in microfluidic devices. In this study, however, the effect of platelet aggregation was negligible: when the MPV values of the retentate and filtrate samples (weighted by their respective volumetric outputs) are combined, there is no significant difference with respect to the input PRP samples (Fig. 5b). We also tested the effects of shear on the platelet population by measuring P-selectin expression, and found that the filtrate sample typically showed slightly *lower* levels of P-selectin expression than the input PRP (Fig. 5c). The weighted result in this case (Fig. 5d), however, suggests that the increased P-selectin expression of platelets in the centre channel (retentate) is slightly higher than would be explained by retention of existing activated platelets and platelet aggregates alone.

Results from additional measurements to characterize the extent of platelet activation, before and after being processed by CIF devices, are presented in Supplementary Table 1. Surface phosphatidylserine (PS) exposure (quantified via Annexin V binding) and integrin αIIbβ3 activation (quantified via PAC1 binding) were studied in (n = 5) PRP samples run through the same devices (OR-7, RS-7/8/9) and at the same driving pressures (6.25, 12.5, and 25 PSI) as in Fig. 5. PS exposure in retentate output samples in nearly all cases (except the 12.5 PSI for the RS-8 device) was significantly higher than corresponding filtrate data (p < 0.05), and the filtrate data were very similar to the input PRP sample measurements, mirroring the trend seen in the MPV and P-selectin measurements. PAC1 binding measurements suggest that neither device type nor driving pressure has a significant effect on αIIbβ3 activation of platelets recovered from the centre (retentate) or side (filtrate) outlets of the devices, as compared to that of the input PRP samples.

## Discussion

This study demonstrates, for the first time, a microfluidic device capable of highly efficient removal of leukocytes from undiluted PRP with platelet recovery and volumetric throughput sufficiently high to suggest the feasibility of using microfluidics in practical, full-scale leukoreduction (LR) applications. When LR is performed by blood centres and hospitals on donated units of PRP, removal of approximately 99.7–99.9% of leukocytes (2.5–3.0 log LR) would typically produce residual leukocyte counts that would satisfy FDA requirements. Similarly, 85% platelet recovery is another FDA standard that leukocyte reduction filters must consistently pass^8^. Our data show that the CIF-based devices described in this study could remove 99–99.9% of leukocytes (2–3 log LR) from undiluted PRP (Fig. 4b) while recovering >80% of platelets (Fig. 4c) at a volumetric throughput of >0.75 mL/min (Fig. 4a). For instance, the RS-7 device produced, on average, >3 log LR with >80% platelet recovery at a flowrate of ~0.8 mL/min. Multiplexing several such CIF devices in parallel (to match the volumetric throughput of conventional LR filters) will enable a direct comparison with existing technology, and is the subject of our follow-on study.

The maximal driving pressures/flowrates (Fig. 4a) we were able to use in this study were limited by the deformation of features of microfluidic devices made of PDMS (effects of these deformations on the performance of other particle separation approaches have been well-documented). Eventual manufacture of the CIF devices described here in rigid thermoplastic should enable a similar level of LR performance at significantly higher flowrates.

Our data from RS-CIF devices with different c.d. (RS-7, -8 and -9) highlights the trade-off between the degree of LR and platelet recovery for any size-based separation. Collecting the maximum amount of platelets possible from donated PRP, however, may not necessarily produce the highest quality platelet product. Given that there is a natural, low level of baseline platelet activation *in vivo* – which is then exacerbated by the drawing and processing of blood – it is potentially advantageous to remove from donated PRP not only the unwanted leukocytes, but also any aggregates of activated platelets. Our data show that platelets remaining in the retentate output of CIF devices are significantly larger and typically more activated than those in the input sample, while the filtrate platelets are slightly smaller, show less P-selectin expression than the input PRP (Fig. 5), and are not elevated in PS exposure nor αIIbβ3 activation (Supplementary Table 1). These results suggest that LR of PRP in this relatively gentle, flow-through manner could produce a platelet product potentially superior to that of conventional LR filters, which do not remove platelet aggregates.

The maximum nominal shear rate within our CIF devices (Fig. 2) was much higher than is typically required to cause platelet activation *in vivo* or *in vitro*^35^,^36^,^37^,^38^,^39^,^40^. It is likely however that platelets passed through the regions of maximal shear (typically along the edges of the posts in the centre channel) so quickly that the effective exposure to shear stress within our system remained below what was necessary to cause measurable platelet activation, even at the highest driving pressures (Fig. 5c,d). Only the filtrate of the RS-9 device, the filtrate of the OR-7 device, and the retentate of the RS-9 device showed a slight increase in either P-selectin expression, PS-exposure, and PAC1 binding respectively, with elevated driving pressure (Fig. 5c, open squares, and Supplementary Table 1). These isolated results, however, are most likely due to factors other than increased flow rate leading to increased shear, as the OR-7 device exhibits a maximum shear rate several fold higher than any of the RS devices (Fig. 2b). The slight increase in cumulative P-selectin expression seen for all devices (Fig. 5c,d) may, therefore, represent the negative effects from the contact of platelets with PDMS and/or tubing materials, and subsequent collection into open-air vials, rather than any effects attributable to shear stress.

The CIF approach has several important advantages over alternative microfluidic methods used for particle separation. Other devices based on co- or cross-flow filtration (which may appear superficially similar to CIF) typically employ simple size exclusion to separate/concentrate larger particles. For example, Sethu *et al.* demonstrated removal of >97% of leukocytes (>1.5-log LR) from whole blood in a co-flow filtration device with exceedingly small (2.5 μm) gaps and very low (~5 μL/min) volumetric throughput^24^. Although these specific results are limited by the presence of a large number of RBCs in WB, such a level of performance – and use of exceedingly small device features – suggests this approach is insufficient for any practical PRP LR application as well.

Devices based on deterministic lateral displacement (DLD) typically have excellent resolution making them particularly well-suited for size-based separation of blood cells. A DLD device capable of removing ~98% of leukocytes from whole blood with a 115 μL/min/atm throughput has been demonstrated previously, which is only marginally below the performance of the CIF devices demonstrated in this study (99–99.9% leukocyte removal at ~440 μL/min/atm throughput), although they did not exceed 3 PSI driving pressure, to avoid deformation effects. In a different application focused on isolating circulating tumour cells (CTCs), multiplexing several rigid DLD devices (made out of silicon and glass, within a pressure-supporting external manifold) allowed for the capture of 85% of CTCs from a diluted blood sample at an impressive throughput of 2.5 mL/min/atm. That device would not, however, be able to perform LR of PRP because of its significantly larger c.d. (CTCs are much larger than the smallest leukocytes) and a filtration ratio of mere 4:1 (compared to 15:1 for CIF devices in this study).

The most significant limitation of both the size exclusion and the DLD approaches is their inherent reliance on very small features. The foundation of DLD approach is the ‘bumping’ of particles above a certain size threshold in a direction normal to the fluid flow by a series of posts that shift laterally along the length of the device. The posts are typically separated by a flow channel ‘gap’ that is a few times larger than the particles of interest. However, once the posts have shifted a given amount, a new post must invariably ‘emerge’ from the sidewall of the device, and gradually move away from it with each row, until a complete new gap is formed (e.g. see Fig. 1 of Inglis *et al.*^23^). For the particle separation to work properly, the DLD approach requires these very narrow (sub gap size) features to be reproduced precisely, which may explain why DLD devices are often fabricated as precisely-etched silicon wafers bonded to glass covers (rather than with the simpler ‘soft lithography’ techniques we have employed here)^20^,^21^,^41^. All existing practical methods for manufacturing microchannel-based devices are constrained by a maximum aspect ratio of: (a) the size of the part’s smallest feature, to (b) how deep the channels can be while still allowing for the part to be demoulded without narrow features bending or breaking off, nor narrowly-spaced features bleeding or sticking together. Thus, the very small features required by both steric filtration and DLD approaches, in addition to directly limiting manufacturability, also reduce their maximum achievable volumetric throughput by limiting the depth of the channels that can be fabricated. The need to overcome these fundamental limitations imposed by the minimum feature size/spacing has been recently driving the development of alternative separation methods to the DLD approach^27^.

The minimal feature size for the next-generation RS-CIF devices described in this study was 16 μm (i.e. the size of the inter-post gap, Fig. 1), which is substantially larger than would be required for either a DLD or size exclusion device. We overcame the need for using very fine features in design of our next-generation CIF devices by introducing an initial series of progressively-shortening side channel segments with widths larger than the inter-post gap (Fig. 1a). Importantly, within the CIF framework, the inter-post gap size is a user-controlled parameter that can be made larger, as needed, while allowing design of a still fully-functioning (albeit correspondingly lengthier) device.

Similar to the CIF approach, the high-speed particle size-separation technique of ‘inertial focusing’ is not limited by the minimum feature size, which gives it great utility in many practical applications. However, the well-documented difficulty in designing devices with predictable performance, and the high sensitivity of such devices to variations in both flowrate and inlet particle concentration, are two major drawbacks of inertial focusing. For example, while very dilute samples may show ~95% leukocyte removal at an optimal flowrate, either halving or doubling the flowrate can cause the separation efficiency to drop below 80%. In contrast, the efficiency of leukocyte removal by CIF devices in this study remained above 99.9% for the RS-7 device and >99% for the RS-8 device (Fig. 4), even when operated at flow rates well below or similar to those optimal for certain inertial focusing devices (e.g. see Fig. 5 of Nivedita and Papautsky^42^). Importantly, CIF based devices can perform separation even when using fluids with particle volume fractions far above what is typically limiting for inertial focusing (i.e. 1–3% v/v).

In summary, the next generation CIF approach described in this study was able to overcome many of the drawbacks of other microfluidic methods and achieve macro-scale flowrates – without using excessively high driving pressure and while still maintaining outstanding particle separation performance (e.g. >99.3% leukocyte reduction and >86% platelet recovery at a flowrate of ~1 mL/min, with the RS-8 device at 25 PSI). Our data demonstrate the feasibility of using a CIF-based microfluidic device for leukoreduction of platelet rich plasma. Further development of this technology could enable an entirely novel approach to PRP leukoreduction that could potentially produce higher quality platelets at lower cost, benefiting millions of patients receiving platelet transfusions every year worldwide.

## Materials and Methods

### Device design and fabrication

Original CIF device design and fabrication methods have been described previously. Briefly, CAD device designs were transferred from chrome-on-glass photomasks (Photo Sciences, Inc., Torrance, CA) into photoresist (SU8 3050; MicroChem Corp, Newton, MA) spun onto 4′′ silicon wafers (University Wafer, South Boston, MA) using UV (i-line) exposure (ETI/6/350/NUV/DCCD/M mask aligner, Evergreen Technology Inc, San Jose, CA). The 135–150 μm deep structures were created by sequentially applying two layers of the photoresist. Following exposure and development of the photoresist, wafers were treated with (tridecafluoro-1,1,2,2-tetrahydrooctyl) trichlorosilane (CAS# 78560-45-6, Gelest Inc, Morrisville, PA) under vacuum for 24 hours.

A custom 7.5:1 (base: crosslinker) poly(dimethylsiloxane) (PDMS) mixture (SylGard 184, Dow Corning Corp, Midland, MI) was used to replicate the master wafer. The PDMS replicas were bonded to 10:1 PDMS-coated Petri dishes using oxygen plasma (Plasmalab 80 Plus, Oxford Instruments, Abingdon, United Kingdom). Bonded devices were treated with 1% (w/v) aqueous solution of mPEG-silane (MW 5000, Laysan Bio Inc, Arab, AL) for 30 minutes, followed by GASP buffer (9 mM, Na_2_HPO_4_, 1.3 mM NaH_2_PO_4_, 140 mM NaCl, 5.5 mM glucose, 1% w/v bovine serum albumin, 290 mmol kg^−1^, pH 7.4) for a minimum of one hour, before use.

### Calculations for patterning reduced-shear CIF device designs

In order to maintain a consistent c.d. along the length of an RS-CIF device, the value of *f*_*gap*_ can no longer be kept constant and must be scaled relative to the progressive narrowing of the device’s centre channel (e.g. see Fig. 3 of Gifford *et al.*^34^) We have assumed, similar to the previous work of others in the field, that (a) the velocity profile of the centre channel in cross-section is approximately parabolic, and (b) the width of the streamline that is diverted into a side channel at each (filtration) gap is roughly proportional to the effective c.d. of the device at that gap. The filtration fraction at each gap, *f*_*gap*_(*i*), for an RS-CIF-based device is then given by eqn. (1), where *w*_*c*_(*i*) is the width of the centre channel at gap *i*, and *w*_*c*_(*ref*) and *f*_*gap*_(*ref*) are reference values known to produce the desired c.d. of a corresponding OR-CIF device.


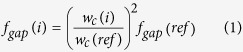


OR-7 was patterned using *w*_*c*_ = 100 μm and *f*_*gap*_ = 5.8 × 10^−4^, a combination of parameters we found corresponding to a c.d. of 7 μm previously^34^. Using these as references values, the RS-7 device with an approximately-equivalent c.d. of 7 μm was patterned by starting with a centre channel of width *w*_*c*_(*i* = *0*) = 300 μm, and an *f*_*gap*_(*i*) value calculated using eqn. (1). The widths of the centre and side channels, *w*_*c*_(*i*) and *w*_*s*_(*i*), were then calculated for the subsequent gaps to maintain a constant maximum shear rate in the centre channel, while also syphoning the appropriate fraction of fluid out of the centre channel, until the width of the centre channel reached a minimum allowed value (set to 150 μm in this study to reduce the effects of any potential clumping of cells in the centre channel). After the minimum allowed value was reached, *w*_*c*_(*i*) was kept constant and only *w*_*s*_(*i*) increased, until the desired final ratio of side to centre channel width (i.e. total amount of filtration) was reached.

### Sample preparation and device operation

All experimental protocols involving human blood samples were approved by the University of Houston Institutional Review Board (Committee for the Protection of Human Subjects 1). Informed consent was obtained from all subjects. All experiments were performed in accordance with guidelines and regulations established by the University of Houston and the U.S. Department of Health and Human Services for the protection of human study subjects. Whole blood (WB) was obtained from healthy consenting volunteers via venepuncture (8.5 mL ACD Vacutainer tubes, BD Biosciences, Franklin Lakes, NJ). WB from each collection tube was transferred into a 10 mL syringe, inverted, and allowed to sediment at unit gravity for 3 hours. Unit gravity separation was used to take advantage of rouleaux formation in order to further exaggerate the existing sedimentation velocity differences between platelets and RBCs, enabling a more effective extraction of PRP from the original whole blood samples. The supernatant PRP (~2–3 mL) from 6–10 syringes were collected into a 30 mL syringe and gently mixed.

The PRP sample was driven into the device inlet through 0.51 mm I.D. Tygon tubing (Cole-Palmer, Vernon Hills, IL) using a driving pressure of 6.25, 12.5 and 25 PSI (equal to 43, 86, and 172 kPa, respectively) created by applying the appropriate weight directly to the wings of the inverted 30 mL syringe, while clamped to the lab bench. Output samples were collected through 0.86 mm I.D. (side channel) and 0.25 mm I.D. (centre channel) tubing (Scientific Commodities, Havasu City, AZ) into polypropylene microcentrifuge tubes. The devices were operated until ~1 mL of the PRP sample had passed through. The device operation time and the sample volume from each outlet were recorded.

### Sample analysis

The platelet concentration, mean platelet volume (MPV), and leukocyte concentration for all samples were measured with a haematology analyser (Medonic M-Series, Boule Diagnostics Int AB, Stockholm, Sweden). The very low leukocyte count in samples collected from the side (filtrate) channels were measured using Leucocount™ Kit on a FACS Aria II flow cytometer (BD Biosciences, Franklin Lakes, NJ) following manufacturer’s protocol. Briefly, after collection, 100 μl of the filtrate sample was added to a BD Trucount tube, followed by 400 μL of BD Leucocount reagent, then gently vortexed for 1 sec, incubated for 5 minutes in the dark at room temperature, and then subjected to flow cytometric data acquisition.

Platelet P-selectin expression, PS exposure, and PAC1 binding were also measured on the FACS Aria II flow cytometer. Samples for P-selectin expression were prepared by incubating ~10^6^ platelets with 2 μL of CD42b-PerCP (HIP1) and either 2 μL (AK-4) CD62p (P-selectin) -FITC (samples) or 2 μL (m2b-25G4) IgG2b-FITC (isotype control) for 20 minutes in 100 μL of PBS (pH 7.4)^43^. Both markers were purchased from eBioscience (San Diego, CA). Platelet samples were then fixed in 1.5 mL PBS with 0.25% w/v paraformaldehyde and 0.5% w/v bovine serum albumin, pH 7.4, and FC analysed within one hour of fixation.

The PAC1 binding assay samples were prepared by incubating ~10^6^ platelets with 2 μL of CD42b-PerCP (HIP1) and 20 μL Anti-Human PAC1-FITC (BD Biosciences, Franklin Lakes, NJ) in 100 μL of PBS (pH 7.4). A sample that also included 10 μL RGDS solution (10 mg/mL in PBS) was used as negative control, as RGDS peptide competitively inhibits PAC-1 binding^44^. After incubation for 20 minutes, the samples were fixed in 1.5 mL PBS with 0.25% w/v paraformaldehyde and 0.5% w/v bovine serum albumin, pH 7.4, and FC analysed within one hour of fixation.

To measure PS exposure on the platelet surface, samples were prepared by incubating ~10^6^ platelets with 4 μL of Alexa 488-labelled Annexin V and 2 μL of CD42b-PerCP (HIP1) at room temperature for 20 minutes in 100 μL of N-(2-hydroxyethyl)piperazine-N′-2-ethanesulfonic acid (HEPES)-buffered saline (10 mmol/L HEPES, 140 mmol/L NaCl, pH 7.4) containing either 2.5 mmol/L calcium or 5 mmol/L K2-ethylenediaminetetraacetate (K2-EDTA). The latter served as a negative control to exclude the nonspecific binding between Annexin V and PS^45^. After incubation, the samples were mixed with 1 mL of the appropriate buffer and were analysed on FC immediately.

To obtain dual-stained images of leukocytes and platelets within the device (for Supplementary Figure 1), platelets were first stained and incubated for 30 minutes using a 1:1:1 ratio of PRP, PBS (pH 7.4) and Anti-Human CD41 BV421 (BD Biosciences, Franklin Lakes, NJ). The sample was then washed by removing the supernatant following centrifugation at 200 g for 10 minutes, with the cells then resuspended in PBS. After the third wash, plasma was used to resuspend the cells for imaging. To stain the leukocytes, SYTO-16 green fluorescent nucleic acid stain (ThermoFisher, Waltham, MA), final concentration 10 μM, was then added and incubated for 30 minutes in the dark. For those image/videos with fluorescently stained leukocytes only, SYTO-16 dye was added (final concentration 10 μM) directly to the PRP and incubated for 30 minutes in the dark. Images and videos were captured using an inverted microscope (IX71, Olympus America Inc., Center Valley, PA) with a digital CMOS camera (ORCA-Flash4.0, Hamamatsu, Bridgewater, NJ). Fluorescence (X-Cite^®^ 120LED, Lumen Dynamics, Ontario, Canada) was combined with ET - EGFP (FITC/Cy2) filter or ET-DAPI filter (both filters are from Chroma Technology Corp, Bellows Falls, VT) for excitation.

### Statistical analysis

For all devices, PRP samples from n = 5 volunteers were tested. Statistical significance in paired data between samples from different outlets on the same device (p < 0.05), and between samples from different devices or driving pressures (p < 0.01), were calculated using two-tailed paired Student’s *t*-test.

### Computational fluidic dynamics simulations

CFD modelling was performed to investigate shear rates within devices using finite element analysis software COMSOL (COMSOL, Inc., Burlington, MA). CFD simulations were carried out using the mesh with 267 μm maximum and 6.7 μm minimum element size. No-slip boundary conditions, Newtonian fluid, and laminar flow were assumed. Volumetric flow rates from 25 PSI experiments were used for each device.

## Additional Information

**How to cite this article**: Xia, H. *et al.* A high-throughput microfluidic approach for 1000-fold leukocyte reduction of platelet-rich plasma. *Sci. Rep.* **6**, 35943; doi: 10.1038/srep35943 (2016).

## Supplementary Material

Supplementary Information

Supplementary Video 1

Supplementary Video 2

Supplementary Video 3

## References

[b1] Heal J. M. & Blumberg N. Optimizing platelet transfusion therapy. Blood reviews 18, 149–165 (2004). 15183900 10.1016/S0268-960X(03)00057-2

[b2] Guidance for Industry: Pre-Storage Leukocyte Reduction of Whole Blood and Blood Components Intended for Transfusion. *U.S. Food and Drug Administration Guidances* http://www.fda.gov/BiologicsBloodVaccines/GuidanceComplianceRegulatoryInformation/Guidances/Blood/ucm320636.htm (2012).

[b3] Paglino J. C., Pomper G. J., Fisch G. S., Champion M. H. & Snyder E. L. Reduction of febrile but not allergic reactions to RBCs and platelets after conversion to universal prestorage leukoreduction. Transfusion 44, 16–24 (2004). 14692962 10.1046/j.0041-1132.2004.00608.x

[b4] Heal J., Masel D., Rowe J. & Blumberg N. Circulating immune complexes involving the ABO system after platelet transfusion. British journal of haematology 85, 566–572 (1993). 8136280 10.1111/j.1365-2141.1993.tb03349.x

[b5] Bowden R. A. *et al.* A comparison of filtered leukocyte-reduced and cytomegalovirus (CMV) seronegative blood products for the prevention of transfusion-associated CMV infection after marrow transplant [see comments]. Blood 86, 3598–3603 (1995). 7579469

[b6] Blumberg N. *et al.* An association between decreased cardiopulmonary complications (transfusion‐related acute lung injury and transfusion‐associated circulatory overload) and implementation of universal leukoreduction of blood transfusions. Transfusion 50, 2738–2744 (2010). 20561296 10.1111/j.1537-2995.2010.02748.x PMC2944002

[b7] Hart S., Cserti‐Gazdewich C. & McCluskey S. Red cell transfusion and the immune system. Anaesthesia 70, 38–e16 (2015). 25440393 10.1111/anae.12892

[b8] Fung M. AABB technical manual, 18th edn (AABB press, Bethesda, MD, 2014).

[b9] Dzik S. Leukodepletion blood filters: filter design and mechanisms of leukocyte removal. Transfusion Medicine Reviews 7, 65–77 (1993). 8481601 10.1016/s0887-7963(93)70125-x

[b10] Bruil A., Beugeling T., Feijen J. & Aken W. G. The mechanisms of leukocyte removal by filtration. Transfusion medicine reviews 9, 145–166 (1995). 7795332 10.1016/s0887-7963(05)80053-7

[b11] Sharma R. & Marwaha N. Leukoreduced blood components: Advantages and strategies for its implementation in developing countries. Asian journal of transfusion science 4, 3 (2010). 20376259 10.4103/0973-6247.59384 PMC2847337

[b12] Monroy R., Cook D., Ogier W. & Schmittling R. Inventors; Eligix, Inc., assignee. Whole blood separator apparatus and method of use. United States Patent Application 20020058030. 2002 May 16.

[b13] Elias M. *et al.* *In vitro* evaluation of a high-efficiency leukocyte adherence filter. Annals of hematology 63, 302–306 (1991). 1756191 10.1007/BF01709651

[b14] Wadhwa M. *et al.* Cytokine levels as performance indicators for white blood cell reduction of platelet concentrates. Vox sanguinis 83, 125–136 (2002). 10.1046/j.1423-0410.2002.00203.x 12201842

[b15] Ferrer F., Rivera J., Corral J., González-Conejero R. & Vicente V. Evaluation of Leukocyte–Depleted Platelet Concentrates Obtained by In–Line Filtration. Vox sanguinis 78, 235–241 (2000). 10895097 10.1159/000031187

[b16] Devine D. *et al.* Effects of prestorage white cell reduction on platelet aggregate formation and the activation state of platelets and plasma enzyme systems. Transfusion 39, 724–734 (1999). 10413280 10.1046/j.1537-2995.1999.39070724.x

[b17] O’brien J. & Salmon G. Shear stress activation of platelet glycoprotein IIb/IIIa plus von Willebrand. Blood 70, 1354–1361 (1987). 3499187

[b18] Han K.-H. & Frazier A. B. Lateral-driven continuous dielectrophoretic microseparators for blood cells suspended in a highly conductive medium. Lab on a Chip 8, 1079–1086 (2008). 18584082 10.1039/b802321b

[b19] Hou H. W. *et al.* Microfluidic devices for blood fractionation. Micromachines 2, 319–343 (2011).

[b20] Huang R. *et al.* A microfluidics approach for the isolation of nucleated red blood cells (NRBCs) from the peripheral blood of pregnant women. Prenatal diagnosis 28, 892–899 (2008). 18821715 10.1002/pd.2079 PMC4482103

[b21] Karabacak N. M. *et al.* Microfluidic, marker-free isolation of circulating tumor cells from blood samples. Nature protocols 9, 694–710 (2014). 24577360 10.1038/nprot.2014.044 PMC4179254

[b22] Loutherback K. *et al.* Deterministic separation of cancer cells from blood at 10 mL/min. AIP advances 2, 042107 (2012). 10.1063/1.4758131 PMC3477176 23112922

[b23] Inglis D. W., Lord M. & Nordon R. E. Scaling deterministic lateral displacement arrays for high throughput and dilution-free enrichment of leukocytes. Journal of Micromechanics and Microengineering 21, 054024 (2011).

[b24] Sethu P., Sin A. & Toner M. Microfluidic diffusive filter for apheresis (leukapheresis). Lab on a Chip 6, 83–89 (2006). 16372073 10.1039/b512049g

[b25] Chen X., Liu C. C. & Li H. Microfluidic chip for blood cell separation and collection based on crossflow filtration. Sensors and Actuators B: Chemical 130, 216–221 (2008).

[b26] Yamada M. & Seki M. Hydrodynamic filtration for on-chip particle concentration and classification utilizing microfluidics. Lab on a Chip 5, 1233–1239 (2005). 16234946 10.1039/b509386d

[b27] Inglis D. W. & Herman N. A scalable approach for high throughput branch flow filtration. Lab on a chip 13, 1724–1731 (2013). 23493870 10.1039/c3lc50192b

[b28] VanDelinder V. & Groisman A. Separation of plasma from whole human blood in a continuous cross-flow in a molded microfluidic device. Analytical chemistry 78, 3765–3771 (2006). 16737235 10.1021/ac060042r

[b29] Shevkoplyas S. S., Yoshida T., Munn L. L. & Bitensky M. W. Biomimetic autoseparation of leukocytes from whole blood in a microfluidic device. Analytical chemistry 77, 933–937 (2005). 15679363 10.1021/ac049037i PMC3022340

[b30] Zheng S., Liu J.-Q. & Tai Y.-C. Streamline-based microfluidic devices for erythrocytes and leukocytes separation. Microelectromechanical Systems, Journal of 17, 1029–1038 (2008).

[b31] Martel J. M. *et al.* Continuous Flow Microfluidic Bioparticle Concentrator. Scientific reports 5 (2015). 10.1038/srep11300 PMC4462155 26061253

[b32] Wu Z., Chen Y., Wang M. & Chung A. J. Continuous inertial microparticle and blood cell separation in straight channels with local microstructures. Lab on a Chip (2016). 10.1039/c5lc01435b 26725506

[b33] Cupelli C. *et al.* Leukocyte enrichment based on a modified pinched flow fractionation approach. Microfluidics and nanofluidics 14, 551–563 (2013).

[b34] Gifford S. C., Spillane A. M., Vignes S. M. & Shevkoplyas S. S. Controlled incremental filtration: a simplified approach to design and fabrication of high-throughput microfluidic devices for selective enrichment of particles. Lab Chip 14, 4496–4505 (2014). 25254358 10.1039/c4lc00785a PMC4247995

[b35] Sakariassen K. S. *et al.* Shear-induced platelet activation and platelet microparticle formation in native human blood. Thrombosis research 92, S33–S41 (1998). 9886908 10.1016/s0049-3848(98)00158-3

[b36] Reininger A. J. *et al.* Mechanism of platelet adhesion to von Willebrand factor and microparticle formation under high shear stress. Blood 107, 3537–3545 (2006). 16449527 10.1182/blood-2005-02-0618 PMC1895770

[b37] Holme P. A. *et al.* Shear-induced platelet activation and platelet microparticle formation at blood flow conditions as in arteries with a severe stenosis. Arteriosclerosis, thrombosis, and vascular biology 17, 646–653 (1997). 10.1161/01.atv.17.4.646 9108776

[b38] Tangelder G., Slaaf D. W., Arts T. & Reneman R. S. Wall shear rate in arterioles *in vivo*: least estimates from platelet velocity profiles. American Journal of Physiology-Heart and Circulatory Physiology 254, H1059–H1064 (1988). 10.1152/ajpheart.1988.254.6.H1059 3381893

[b39] Laurent P.-A. *et al.* Platelet PI3Kβ and GSK3 regulate thrombus stability at a high shear rate. Blood 125, 881–888 (2015). 25398937 10.1182/blood-2014-07-588335

[b40] Dayananda K. M., Singh I., Mondal N. & Neelamegham S. von Willebrand factor self-association on platelet GpIbα under hydrodynamic shear: effect on shear-induced platelet activation. Blood 116, 3990–3998 (2010). 20696943 10.1182/blood-2010-02-269266 PMC2981547

[b41] Huang L. R., Cox E. C., Austin R. H. & Sturm J. C. Continuous particle separation through deterministic lateral displacement. Science 304, 987–990 (2004). 15143275 10.1126/science.1094567

[b42] Nivedita N. & Papautsky I. Continuous separation of blood cells in spiral microfluidic devices. Biomicrofluidics 7, 054101 (2013). 10.1063/1.4819275 PMC3779264 24404064

[b43] Skinner M. P., Lucas C., Burns G., Chesterman C. & Berndt M. C. GMP-140 binding to neutrophils is inhibited by sulfated glycans. Journal of Biological Chemistry 266, 5371–5374 (1991). 1706335

[b44] Shattil S. J., Cunningham M. & Hoxie J. A. Detection of activated platelets in whole blood using activation-dependent monoclonal antibodies and flow cytometry. Blood 70, 307–315 (1987). 3297204

[b45] Albanyan A. M., Murphy M. F., Rasmussen J. T., Heegaard C. W. & Harrison P. Measurement of phosphatidylserine exposure during storage of platelet concentrates using the novel probe lactadherin: a comparison study with annexin V. Transfusion 49, 99–107 (2009). 18954406 10.1111/j.1537-2995.2008.01933.x

