# Author Correction: A high-throughput microfluidic approach for 1000-fold leukocyte reduction of platelet-rich plasma

**DOI:** 10.1038/s41598-022-18449-5

**Published:** 2022-08-29

**Authors:** Hui Xia, Briony C. Strachan, Sean C. Gifford, Sergey S. Shevkoplyas

**Affiliations:** 1 Department of Biomedical Engineering, University of Houston, Houston, TX 77204 USA; 2 Present Address: Present address: Halcyon Biomedical Incorporated, Friendswood, TX 77546 USA

Correction to: *Scientific Reports* 10.1038/srep35943, published online 24 October 2016

This Article contains an error in equation 1, where the terms *w*_*c*_(*i*) and *w*_*c*_(*ref*) are inverted.

$${f}_{gap}\left(i\right)={\left(\frac{{w}_{c}\left(i\right)}{{w}_{c}\left(ref\right)}\right)}^{2}{f}_{gap}(ref)$$

should read:

$${f}_{gap}\left(i\right)={\left(\frac{{w}_{c}\left(ref\right)}{{w}_{c}\left(i\right)}\right)}^{2}{f}_{gap}(ref)$$

