# Supplementary material for: A high-throughput microfluidic approach for 1000-fold leukocyte reduction of platelet-rich plasma
Source: Sci Rep. 2016 Oct 24;6:35943. doi: 10.1038/srep35943 (PMC5075940; doi:10.1038/srep35943)
Supplement: Supplementary Information [file srep35943-s1.pdf]

A high-throughput microfluidic approach for 1000-fold leukocyte reduction of platelet-rich plasma†

Hui Xia, Briony C. Strachan, Sean C. Gifford, and Sergey S. Shevkoplyas*

Supplementary Materials

Supplementary Figure 1

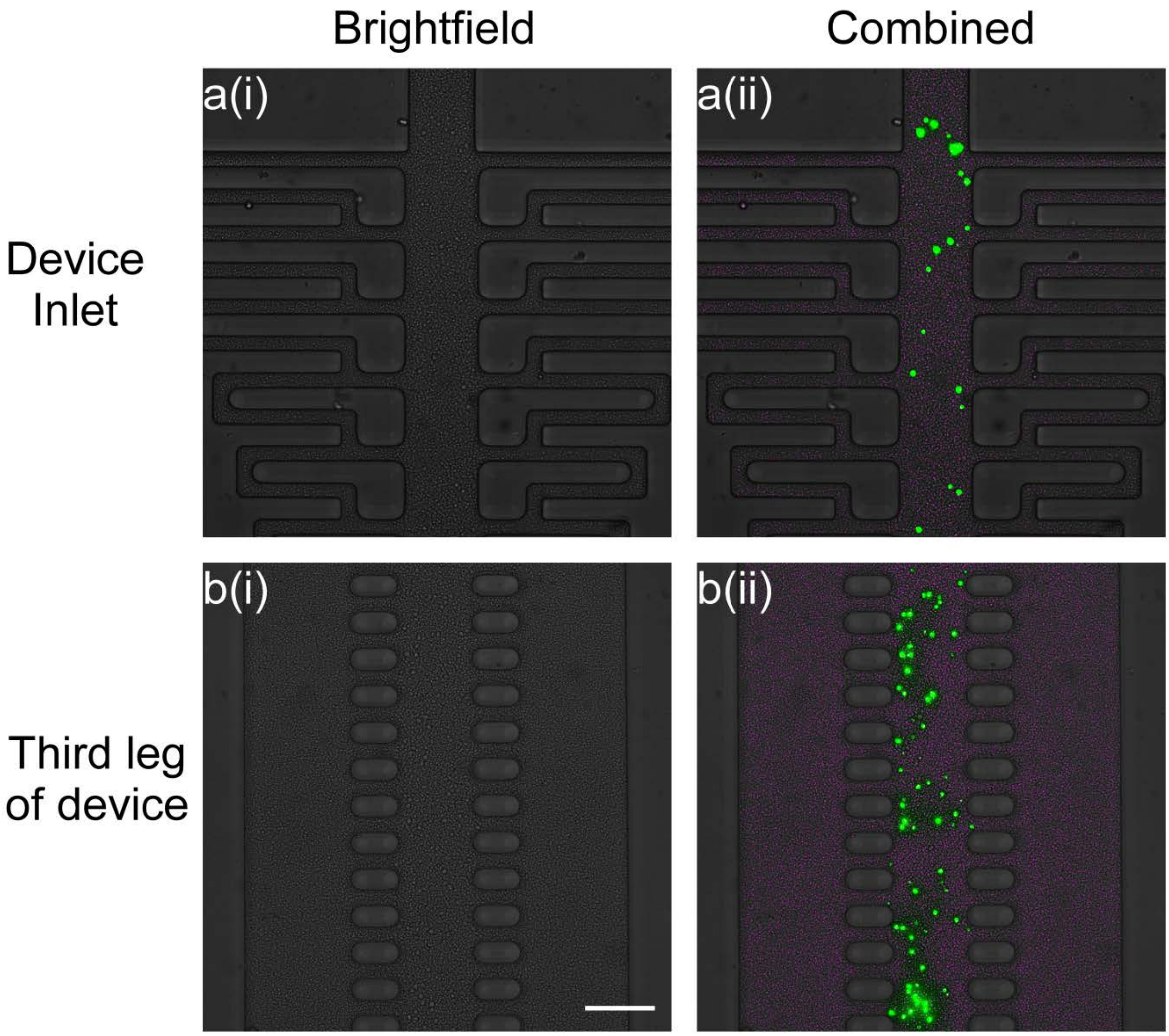

Supplementary Figure 1. Epifluorescence microscopy images of a PRP sample at the inlet (**a**) and third leg (**b**) of an OR-7 CIF device. Leukocytes were labelled with (green) SYTO-16 fluorescent nucleic acid stain and platelets were labelled with (violet) Anti-Human CD41 BV421. Standard brightfield images (**i**) are combined with fluorescent micrographs of leukocytes and platelets (**ii**) to show the representative distribution of the different cell types within the centre (retentate) and side (filtrate) channels of the CIF device. Note not all platelets are stained brightly enough to surpass the intensity level threshold imposed during imaging to allow clear contrast of stained platelets from background fluorescence. Scale bar is 100 μm.

Supplementary Table 1. Levels of PS exposure, PAC1 binding, and P-selectin expression, shown as mean (range) in four CIF devices and under three separate driving pressures. The P-selectin expression data has been demonstrated in **Figure 5** and is presented here for reference.

**Device**	**Driving pressure (PSI)**	**PS Exposure (%)**			**PAC1 binding (%)**			**P-selectin expression (%)**			**P-values**		
		**inlet**	**filtrate**	**retentate**	**inlet**	**filtrate**	**retentate**	**inlet**	**filtrate**	**retentate**	**(1)**	**(2)**	**(3)**
**OR-7**	**6.25**	0.7 (0.5-0.9)	0.6 (0.3-1.6)	2.0 (1.4-3.2)	2.3 (0.7-5.0)	2.5 (1.1-4.1)	2.3 (0.7-4.2)	3.3 (2.3-5.0)	3.2 (1.8-5.1)	8.2 (3.3-11.8)	a,b	NS	a,b
	**12.5**	0.7 (0.5-0.9)	0.4 (0.2-0.6)	1.6 (0.9-2.4)	2.3 (0.7-5.0)	2.5 (0.5-4.4)	2.9 (0.9-6.8)	3.2 (0.8-5.4)	3.2 (0.8-5.9)	6.6 (2.9-9.8)	a,b,c	NS	a
	**25**	0.7 (0.5-0.9)	0.6 (0.4-1.0)	1.7 (1.4-2.1)	2.3 (0.7-5.0)	2.2 (0.9-4.8)	3.1 (1.9-5.9)	3.2 (0.8-4.9)	3.1 (0.9-5.0)	6.9 (2.7-11.2)	a,b	a,b	a,b
**RS-7**	**6.25**	0.5 (0.4-0.9)	0.4 (0.2-0.6)	2.3 (1.0-3.9)	4.0 (1.1-5.7)	4.8 (1.2-8.9)	5.1 (0.9-10.8)	3.7 (1.7-6.0)	3.5 (1.7-5.2)	6.9 (1.8-10.0)	a,b	NS	a,b
	**12.5**	0.5 (0.4-0.9)	0.4 (0.4-0.6)	2.1 (1.6-3.3)	4.0 (1.1-5.7)	5.2 (1.3-9.3)	4.9 (1.1-11.6)	2.6 (1.2-3.9)	2.5 (1.5-4.1)	6.6 (2.1-10.9)	a,b	NS	a
	**25**	0.5 (0.4-0.9)	0.5 (0.4-0.6)	2.3 (1.3-2.9)	4.0 (1.1-5.7)	3.4 (1.0-5.2)	5.2 (1.6-9.2)	3.4 (1.5-4.9)	2.9 (1.6-3.8)	6.5 (1.8-9.1)	a,b	NS	a,b
**RS-8**	**6.25**	0.5 (0.3-0.7)	0.4 (0.3-0.6)	1.7 (0.8-3.3)	3.3 (1.0-4.5)	4.5 (1.2-7.6)	5.4 (2.4-8.1)	2.8 (1.2-4.0)	2.9 (1.4-3.9)	6.0 (2.6-9.2)	a,b	b,d	a,b
	**12.5**	0.5 (0.3-0.7)	0.5 (0.4-0.5)	1.8 (1.2-3.1)	3.3 (1.0-4.5)	4.2 (1.0-6.0)	4.2 (2.0-7.7)	3.1 (1.5-3.9)	2.8 (1.5-3.4)	6.4 (2.6-10.6)	a	b	a,b
	**25**	0.5 (0.3-0.7)	0.7 (0.3-1.1)	2.0 (1.2-2.9)	3.3 (1.0-4.5)	3.9 (1.1-6.3)	3.0 (1.4-5.2)	2.9 (1.6-4.0)	2.9 (1.5-3.9)	6.3 (2.6-9.0)	a,b	NS	a,b
**RS-9**	**6.25**	0.6 (0.3-1.0)	0.5 (0.3-0.7)	2.0 (1.4-2.3)	3.6 (0.7-5.1)	3.2 (0.8-4.8)	3.9 (1.6-6.5)	3.8 (2.6-4.3)	3.2 (1.2-5.5)	6.8 (3.6-9.5)	a,b	NS	a,b
	**12.5**	0.6 (0.3-1.0)	0.5 (0.3-0.8)	2.3 (1.6-3.1)	3.6 (0.7-5.1)	3.0 (1.1-4.2)	3.0 (1.8-4.2)	3.1 (2.0-4.3)	3.1 (2.0-3.8)	6.9 (4.4-9.0)	a,b	NS	a,b,c
	**25**	0.6 (0.3-1.0)	0.8 (0.4-1.6)	2.0 (1.3-2.7)	3.6 (0.7-5.1)	2.6 (1.1-4.9)	2.9 (1.0-4.8)	3.6 (2.2-5.6)	3.7 (2.6-4.6)	7.2 (4.7-10.5)	a,b	NS	a,b

P-values (1) indicates significance of difference ($p<0.05$) between samples from different outlets for PS exposure measurements.
P-values (2) indicates significance of difference ($p<0.05$) between samples from different outlets for PAC1 binding measurements.
P-values (3) indicates significance of difference ($p<0.05$) between samples from different outlets for P-selectin expression measurements.
a denotes $p<0.05$ between the filtrate and retentate; **b** denotes $p<0.05$ between the inlet and the retentate; **c** denotes $p<0.05$ between this driving pressure and the highest driving pressure (25 PSI) in the filtrate; **d** denotes $p<0.05$ between this driving pressure and the highest driving pressure (25 PSI) in the retentate; **NS** denotes $p>0.05$.

Supplementary Video Captions

Supplementary Video 1. Video demonstrating CIF-based leukoreduction of platelet-rich plasma (PRP). PRP, with an initial leukocyte count of $3.6 \times 10^3/\mu L$ is driven through an RS-7 device at a pressure of 6.25 PSI. Fluorescently-labelled leukocytes and (unlabelled) platelets flow into and through the device, with platelets progressively syphoned into its side channels as leukocytes are retained/concentrated in its centre channel. PRP flows past the initial serpentine side segments towards the transition area to pill-shaped posts (**0-18 s**). Leukocytes can be seen to be increasingly concentrated as the PRP progresses through legs 3 and 5 of the device (**21-27 s**), during which its centre channel narrows (to a preset minimum, 150 µm) and its side channels widen. The extent of leukocyte concentration and side channel growth can be observed in the final leg of the device, which is followed to the device exit (**32-62 s**). Focus is partially lost during this section while scanning along the device. At the device outlet, the vast majority of stained leukocytes are seen retained in the centre channel, and far fewer in the side channel (**63-70 s**). Concentrated leukocytes are removed via the centre channel outlet (**74-81 s**), in sharp contrast to the side channels' outlet, which is almost devoid of leukocytes (**86-109 s**).

Supplementary Video 2. Video demonstrating how OR-7 device performance is affected as driving pressure is increased. Platelet-rich plasma, with an initial leukocyte count of $2.6 \times 10^3/\mu L$, is first driven through an OR-7 device at a pressure of 6.25 PSI. In the final leg of the device (shown), fluorescently-labelled leukocytes are seen highly-concentrated in the centre channel, while platelets (unlabelled) have been syphoned into the side channels through each filtration

gap. Few leukocytes can be observed flowing in the side channels of the device. Once the pressure is increased to 12.5 PSI (at **10 s**) several leukocytes can be seen to pass through the gaps, out of the centre channel (where they are desired). This phenomenon appears to be attributable to increased deformation of the soft PDMS structures at higher pressures, which can alter the predicted fluidic streamlines of a CIF device. (See also, **Figure 4b**.)

Supplementary Video 3. Video demonstrating how RS-7 device performance is affected as driving pressure is increased. Platelet-rich plasma, with an initial leukocyte count of 2.5×10^3/µL, is first driven through an RS-7 device at a pressure of 6.25 PSI. As with the OR-7 device (please see **Supplementary Video 2**) few leukocytes can be seen in the RS-7 side channels. Once the pressure is increased to 12.5 PSI (at **10 s**) there is no observable change in the number of leukocytes moving through the filtration gaps into the side channel. The modified design of the reduced-shear device has also minimized the effects of PDMS deformation at higher pressures. (See also, **Figure 4b**.)
